# Supplementary material for: Revisiting the Links-Species Scaling Relationship in Food Webs
Source: Patterns (N Y). 2020 Jul 29;1(7):100079. doi: 10.1016/j.patter.2020.100079 (PMC7660400; doi:10.1016/j.patter.2020.100079)
Supplement: Document S1. Supplemental Experimental Procedures, Figure S1, and Table S1 [file mmc1.pdf]

PATTER, Volume *1*

Supplemental Information

Revisiting the Links-Species Scaling Relationship in Food Webs

Arthur Andrew Meahan MacDonald, Francis Banville, and Timothée Poisot

Supplemental Experimental Procedures

Parameter estimation by Maximum Likelihood

While the full posterior distribution can be sampled using various bayesian machinery, this is not necessary for obtaining point estimates of μ and ϕ. A maximum likelihood estimate of each can be calculated by rearranging eq. 4 and fitting a Beta distribution to the result.

We include this result because ecologists may wish to apply our methods for estimating L, Co or L/S without fitting a Bayesian posterior of their own. This approach loses information about the sample size of webs, but nevertheless provides a close match to both the empirical data and the bayesian posterior.

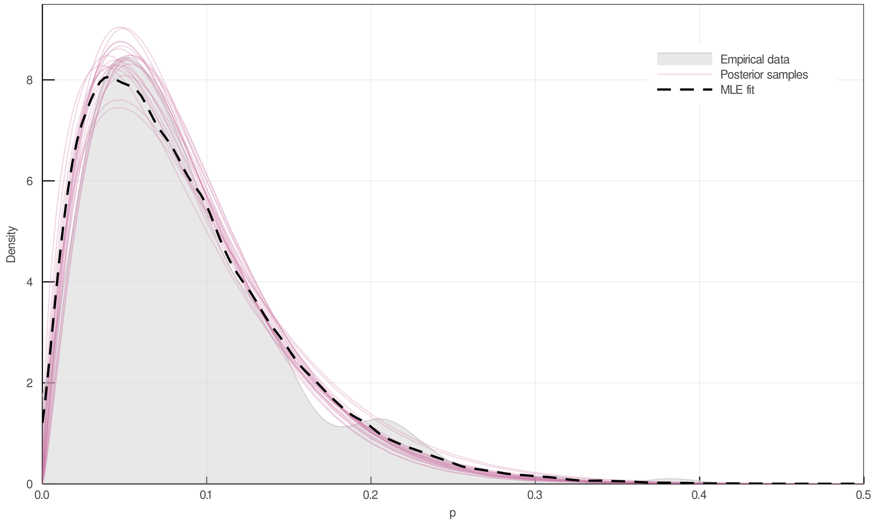

Figure 1: **Figure S1. Parameters can be estimated by Maximum Likelihood.** The maximum likelihood estimate of p is compared to 20 samples from the posterior distribution of the flexible links model. The empirical distribution of p, obtained from all food webs archived on the `mangal.io` database, is also included.

Table S1: Comparison between parameter estimates using maximum likelihood as described above (MLE estimate) and the maximum *a posteriori* values (MAP estimate) as reported in the main text.

parameter	MLE estimate	MAP estimate
μ	0.087	0.086 ± 0.0037

parameter	MLE estimate	MAP estimate
ϕ	21.0	24.3 $\pm$ 2.4
