# Supplementary material for: Revisiting the Links-Species Scaling Relationship in Food Webs
Source: Patterns (N Y). 2020 Jul 29;1(7):100079. doi: 10.1016/j.patter.2020.100079 (PMC7660400; doi:10.1016/j.patter.2020.100079)
Supplement: Document S2. Article plus Supplemental Information [file mmc2.pdf]

Patterns

Article

Revisiting the Links-Species Scaling Relationship in Food Webs

Highlights

- The number of links in a food web scales with the number of species present
- The number of links is also constrained to lie within biologically realistic limits
- We provide a realistic, probabilistic model to predict the number of links
- Our model performs better than previous attempts to predict the number of links

Authors

Arthur Andrew Meahan MacDonald, Francis Banville, Timothée Poisot

Correspondence

a.a.m.macdonald@gmail.com

In Brief

The total number of interactions is a fundamental measure of all types of networks. In food webs, it is strongly associated with the total number of species (nodes). Previous attempts to model the relationship between these quantities made unrealistic predictions, i.e., predicting numbers of links below the minimum set by ecological constraints. Here we provide a realistic and performant model to predict the number of links in a food web from its number of species, taking into account biological constraints.

MacDonald et al., 2020, Patterns *1*, 100079
October 9, 2020 © 2020 The Authors.
https://doi.org/10.1016/j.patter.2020.100079

CellPress

Patterns

CellPress
OPEN ACCESS

Article

Revisiting the Links-Species Scaling Relationship in Food Webs

Arthur Andrew Meahan MacDonald,[1,2,3,*] Francis Banville,[1,2] and Timothée Poisot[1,2]
[1]Département de sciences biologiques, Université de Montréal, Montreal, QC H2V 0B3, Canada
[2]Quebec Centre for Biodiversity Science, Montreal, QC H3A 1B1, Canada
[3]Lead Contact
*Correspondence: a.a.m.macdonald@gmail.com
https://doi.org/10.1016/j.patter.2020.100079

THE BIGGER PICTURE Understanding the functions, stability, resilience, and dynamics of ecological communities requires the investigation of the structure of their networks of interactions. Perhaps the most challenging issue in the study of such networks is that the sampling of ecological interactions is a strenuous task, and as a result, the availability of empirical data is limited. In this contribution we derive a realistic and performant statistical model to predict the number of interactions in a food web from its number of species. Our model could be used as a first-order approximation of network structure. As such, it makes the large-scale study and comparison of ecological networks more accurate and accessible. For instance, the vulnerability of food webs to perturbations could be explored using our model at the regional, continental, or global scales.

1 2 3 4 5 **Proof-of-Concept:** Data science output has been formulated, implemented, and tested for one domain/problem

SUMMARY

Predicting the number of interactions among species in a food web is an important task. These trophic interactions underlie many ecological and evolutionary processes, ranging from biomass fluxes, ecosystem stability, resilience to extinction, and resistance against novel species. We investigate and compare several ways to predict the number of interactions in food webs. We conclude that a simple beta-binomial model outperforms other models, with the added desirable property of respecting biological constraints. We show how this simple relationship gives rise to a predicted distribution of several quantities related to link number in food webs, including the scaling of network structure with space and the probability that a network will be stable.

INTRODUCTION

Community ecologists are fascinated by counting things. It is therefore no surprise that early food web research paid so much attention to counting species, counting trophic links, and uncovering the relationship that binds them, and it is undeniable that these inquiries kick-started what is now one of the most rapidly growing fields of ecology.[1] More species (S) always means more links (L); this scaling is universal and appears both in observed food webs and under purely neutral models of food web structure.[2] In fact, these numbers underlie most measures used to describe food webs.[3] The structure of a food web, in turn, is almost always required to understand how the community functions, develops, and responds to changes,[4,5] to the point where some authors suggested that describing food webs was a necessity for community ecology.[6,7] To this end, a first step is to come up with an estimate for the number of existing trophic links, through sampling or otherwise. Although both L and S can be counted in nature, the measurement of links is orders of magnitude more difficult than the observation of species.[8,9] As a result, we have far more information about values of S. In fact, the distribution of species richness across the world is probably the most frequently observed and modeled ecological phenomenon. Therefore, if we can predict L from S in an ecologically realistic way, we would be in a position to make first-order approximations of food web structure at large scales, even under our current data-limited regime.

Measures of food web structure react most strongly to a handful of important quantities. The first and most straightforward is L, the number of trophic links among species. This quantity can be large, especially in species-rich habitats, but it cannot be arbitrarily large. It is clear to any observer of nature that of all imaginable trophic links, only a fraction actually occur. If an ecological

This is an open access article under the CC BY license (http://creativecommons.org/licenses/by/4.0/).

community contains S species, then the maximum number of links in its food web is S^2: a community of omnivorous cannibals. This leads to the second quantity: a ratio called connectance, defined by ecologists as $Co = L/S^2$. Connectance has become a fundamental quantity for nearly all other measures of food web structure and dynamics.[10] The third important quantity is another ratio, linkage density, $L_D = L/S$. This value represents the number of links added to the network for every additional species in the ecological system. A closely related quantity is $L_D \times 2$, which is the average degree: the average number of species with which any taxa is expected to interact, either as predator or prey. These quantities capture ecologically important aspects of a network, and all can be derived from the observation or prediction of L links among S species.

Because L represents such a fundamental quantity, many predictive models have been considered over the years. Here, we describe three popular approaches before describing our own proposed model. Link-species scaling (LSSL)[11] assumes that all networks have the same average degree; that is, most species should have the same number of links. Links are modeled as the number of species times a constant:

$$L_{LSSL} = b \times S, \qquad \text{(Equation 1)}$$

with $b \approx 2$. This model imagines that every species added to a community increases the number of links by 2—for example, an animal that consumes one resource and is consumed by one predator. This model started to show its deficiencies when data on larger food webs became available: in these larger webs, L increased faster than a linear function of S. Perhaps then all networks have the same connectance?[12] In other words, a food web is always equally filled, regardless of whether it has 5 or 5,000 species. Under the so-called constant connectance model, the number of links is proportional to the richness squared,

$$L_{CC} = b \times S^2, \qquad \text{(Equation 2)}$$

where b is a constant in $]0,1[$ representing the expected value of connectance. The assumption of a scaling exponent of 2 can be relaxed,[12] so that L is not in direct proportion to the maximum number of links:

$$L_{PL} = b \times S^a. \qquad \text{(Equation 3)}$$

This "power law" model can be parameterized in many ways, including spatial scaling and species area relationships.[13] It is also a general case of the previous two models, encompassing both link-species scaling ($a = 1,\ b \approx 2$) and the strict constant connectance ($a = 2,\ 0<b<1$) depending on which parameters are fixed. Power laws are very flexible, and indeed this function matches empirical data well—so well that it is often treated as a "true" model that captures the scaling of link number with species richness,[14–16] and from which we should draw ecological inferences about what shapes food webs. However, this approach is limited, because the parameters of a power law relationship can arise from many mechanisms and are difficult to reason about ecologically.

However, the question of how informative parameters of a power law can be is moot. Indeed, both the general model and its variants share an important shortcoming: they cannot be used for prediction while remaining within the bounds set by ecological principles. This has two causes. First, models that are variations of $L \approx b \times S^a$ have no constraints, with the exception of the "constant connectance" model, in which L_{cc} has a maximum value of S^2. However, we know that the number of links within a food web is both lower and upper bounded:[12,17] there can be no more than S^2 links, and there can be no fewer than $S - 1$ links. This minimum of $S - 1$ holds for food webs in which all species interact—for example, a community of plants and herbivores where no plants are inedible and all herbivores must eat.[12] Numerous simple food webs could have this minimal number of links—for example, a linear food chain wherein each trophic level consists of a single species, each of which consumes only the species below it; or a grazing herbivore that feeds on every plant in a field. Thus the number of links is constrained by ecological principles to be between $S - 1$ and S^2, something which no present model includes. Secondly, accurate predictions of L from S are often difficult because of how parameters are estimated. This is usually done using a Gaussian likelihood for L, often after log transformation of both L and S. While this approach ensures that predicted values of L are always positive, it does nothing to ensure that they stay below S^2 and above $S - 1$. Thus, a good model for L should meet these two needs: a bounded expression for the average number of links as well as a bounded distribution for its likelihood.

Here we suggest a new perspective for a model of L as a function of S that respects ecological bounds and has a bounded distribution of the likelihood. We include the minimum constraint by modeling not the total number of links but the number in excess of the minimum. We include the maximum constraint in a similar fashion to the constant connectance model described above by modeling the proportion of flexible links realized in a community.

Interlude: Deriving a Process-Based Model for the Number of Links

Based on the ecological constraints discussed earlier, we know that the number of links L is an integer such that $S - 1 \leq L \leq S^2$. Because we know that there are at least $S - 1$ links, there can be at most $S^2 - (S - 1)$ links in excess of this quantity. The $S - 1$ minimum links do not need to be modeled, because their existence is guaranteed as a precondition of observing the network. The question our model should address is, therefore, how many of these $S^2 - (S - 1)$ "flexible" links are actually present? A second key piece of information is that the presence of a link can be viewed as the outcome of a discrete stochastic event, with the alternative outcome that the link is absent. We assume that all of these flexible links have the same chance of being realized, which we call p. Then, if we aggregate across all possible species pairs, the expected number of links is

$$L_{FL} = p \times \left[S^2 - (S - 1)\right] + (S - 1), \qquad \text{(Equation 4)}$$

where $p \in [0,1]$. When $p = 1$, L is at its maximum (S^2), and when $p = 0$ it is at the minimum value ($S - 1$). We use the notation L_{FL} to represent that our model considers the number of "flexible" links in a food web; that is, the number of links in excess of the minimum but below the maximum.

Table 1. Comparison of the Four Different Models

Model	Equation	PSIS-LOO	ΔELPD	$SE_{\Delta ELPD}$
Flexible links	Equation 4	2,520.5 ± 44.4	0	0
Power law (Brose et al.[13])	Equation 3	2,564.3 ± 46.6	−21.9	6.5
Constant (Martinez[12])	Equation 2	2,811.0 ± 68.3	−145.3	21.1
Link-species scaling (Cohen and Briand[11])	Equation 1	39,840.1 ± 2,795.1	−18,659.8	1,381.7

The table shows Pareto-smoothed importance sampling values leave-one-out (PSIS-LOO) and their standard deviation. PSIS-LOO is similar to information criteria in that smaller values indicate better predictive performance. Also shown are expected log predictive density (ELPD) differences to the maximum for all models, along with the standard error (SE) of these differences.

Because we assume that every flexible link is an independent stochastic event with only two outcomes, we can follow recent literature on probabilistic ecological networks[18] and represent them as independent Bernoulli trials with a probability of success p. This approach does not capture ecological mechanisms known to act on food webs,[19] but rather captures that any interaction is the outcome of many processes which can overall be considered probabilistic events.[20] The assumption that flexible links can all be represented by Bernoulli events is an appropriate trade-off between biological realism and parameterization requirements.

Furthermore, the observation of L links in a food web represents an aggregation of $S^2-(S-1)$ such trials. If we then assume that p is a constant for all links in a particular food web, but may vary between food webs (a strong assumption that we later show is actually more stringent than what data suggest), we can model the distribution of links directly as a shifted beta-binomial variable:

$$[L|S,\mu,\phi]=\binom{S^2-(S-1)}{L-(S-1)}\frac{B\left(L-(S-1)+\mu\phi,S^2-L+(1-\mu)\phi\right)}{B(\mu\phi,\ (1-\mu)\phi)}, \quad \text{(Equation 5)}$$

where B is the beta function, μ is the average probability of a flexible link being realized (i.e., the average value of p across networks in the dataset), and ϕ is the concentration around this value. The support of this distribution is limited to only ecologically realistic values of L: it has no probability mass below $S-1$ or above S^2. This means that the problem of estimating values for μ and ϕ is reduced to fitting the univariate distribution described in Equation 5. For a more detailed explanation of the model derivation, fitting, and comparison, see Experimental Procedures.

In this paper we will compare our flexible links model with three previous models for L. We estimate parameters and compare the performance of all models using open data from the mangal.io networks database.[21] This online, open-access database collects published information on all kinds of ecological networks, including 255 food webs detailing interactions between consumers and resources.[22] We use these data to show how our flexible links model not only outperforms existing efforts at predicting the number of links but also has numerous desirable properties from which novel insights about the structure of food webs can be derived.

RESULTS AND DISCUSSION

Flexible Links Model Fits Better and Makes a Plausible Range of Predictions

When fit to the datasets archived on mangal.io, all four models fit without any problematic warnings (see Experimental Procedures), while our model for flexible links outperformed previous solutions to the problem of modeling L. The flexible links model, which we fit via a beta-binomial observation model, had the most favorable values of Pareto-smoothed importance sampling values leave-one-out (PSIS-LOO) information criterion (Table 1) and of expected log predictive density (ELPD), relative to the three competing models which used a negative binomial observation model. PSIS serves as a guide to model selection;[23] and like other information criteria it approximates the error in cross-validation predictions. Smaller values indicate a model that makes better predictions. The calculation of PSIS-LOO can also provide some clues about potential model fits; in our case the algorithm suggested that the constant connectance model was sensitive to extreme observations. The ELPD, on the other hand, measures the predictive performance of the model; here, higher values indicate more reliable predictions.[23] This suggests that the flexible links model will make the best predictions of L.

To be useful to ecologists, predictions of L must stay within realistic boundaries determined by ecological principles. We generated posterior predictions for all models and visualized them against these constraints (Figure 1). The LSSL model underestimates the number of links, especially in large networks: its predictions were frequently lower than the minimum $S-1$. The constant connectance and power law models also made predictions below this value, especially for small values of S. The flexible links model made roughly the same predictions but within ecologically realistic values.

The Flexible Links Model Makes Realistic Predictions for Small Communities

Constraints on food web structure are especially important for small communities. This is emphasized in Figure 2, which shows that all models other than the flexible links model fail to stay within realistic ecological constraints when S is small. The link-species scaling model made around 29% of unrealistic predictions of link numbers for every value of S ($3 \leq S \leq 750$). The constant connectance and power law models, on the other hand, also produced unrealistic results but for small networks only: more than 20% were unrealistic for networks comprising less than 12 and 7 species, respectively. Only the flexible links model, by design, never failed

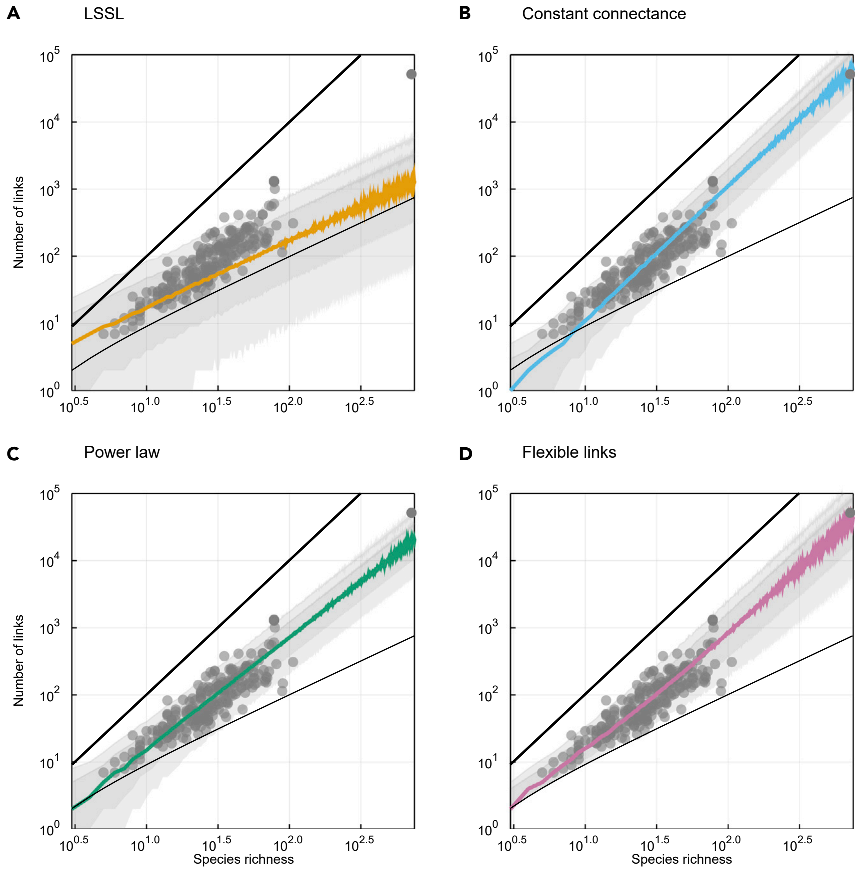

Figure 1. The Flexible Links Model Fits Better and Makes a Plausible Range of Predictions

The number of links is plotted as a function of species richness obtained from the posterior distributions of (A) the link-species scaling, (B) the constant connectance, (C) the power law, and (D) the flexible links models. In each panel, the colored line represents the median predicted link number and the gray areas cover the 78% and 97% percentile intervals. Empirical data from the mangal.io database are plotted in each panel (gray dots), as well as the minimal $S-1$ and maximal S^2 number of links (thinner and bolder black lines, respectively). Predictions from the flexible links model are always plausible: they stay within these biological boundaries.

to predict numbers of links between $S-1$ and S^2. It must be noted that unrealistic predictions are most common in the shaded area of Figure 2, which represents 90% of the empirical data we used to fit the model; therefore, it matters little that models agree for large S, since there are virtually no such networks observed.

Parameter Estimates for All Models

Although we did not use the same approach to parameter estimation as previous authors, our approach to fitting these models recovered parameter estimates that are broadly congruent with previous works. We found a value of 2.2 for b of the LSSL model (Table 2), which is close to the original value of approximately 2.[11] Similarly, we found a value of 0.12 for b of the constant connectance model, which was consistent with original estimates of 0.14.[12] Finally, the parameter values we found for the power law were also comparable with earlier estimates.[13] All of these models were fit with a negative binomial observation model, which has an additional parameter, κ, which is sometimes called a "concentration" parameter. This value increases from the top of our table to the bottom, in the same sequence as predictive performance improves in Table 1. This indicates that the model predictions are more concentrated around the mean predicted by the model (Table 2, column 1).

Our parameter estimates for the flexible links model are ecologically meaningful. For large communities, our model should behave similarly to the constant connectance model and so it is no surprise that μ was about 0.09, which is close to our value of 0.12 for constant connectance. In addition, we obtained a rather large value of 24.3 for ϕ, which shrinks the variance around the mean of p to approximately 0.003 ($\text{var}(p) = \mu(1-\mu)/(1+\phi)$). This indicates that food webs are largely similar in their probability of flexible links being realized (thus showing how our previous assumption that p might vary between food webs to be more conservative than strictly required). The flexible links model also uses fewer parameters than the power law model and makes slightly better predictions, which accounts for its superior performance in model comparison (Table 1). In Figure S1, we compare the maximum *a posteriori* (MAP) estimates of our model parameters with their maximum likelihood estimates.

Connectance and Linkage Density Can Be Derived from a Model for Links

Of the three important quantities that describe networks (L, Co, and L_D), we have directly modeled L only. However, we can use the parameter estimates from our model for L to parameterize a distribution for connectance (L/S^2) and linkage density (L/S). We can derive this by noticing that Equation 4 can be rearranged to show how Co and L_D are linear transformations of p:

$$\text{Co} = \frac{L}{S^2} = p\left(1 - \frac{S-1}{S^2}\right) + \frac{S-1}{S^2} \quad \text{(Equation 6)}$$

and

$$L_D = \frac{L}{S} = p\left(S - \frac{S-1}{S}\right) + \frac{S-1}{S}. \quad \text{(Equation 7)}$$

For food webs with many species, these equations simplify: Equation 4 can be expressed as a second-degree polynomial, $L_{FL} = p \times S^2 + (1-p) \times S + (p-1)$, whose leading term is $p \times S^2$. Therefore, when S is large, Equations 6 and 7 respectively approach $\text{Co} = L/S^2 \approx p$ and $L_D = L/S \approx pS$. A study of Equations 6 and 7 also provides insight into the ecological interpretation of the parameters in our equation. For example, Equation 7 implies

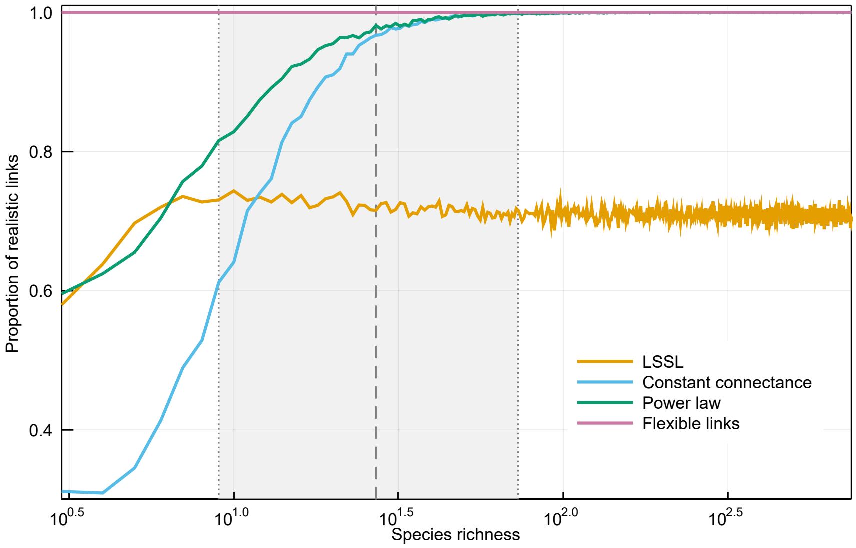

Figure 2. Only the Flexible Links Model Makes Realistic Predictions for Small Communities

Here we show the proportion of posterior predictions from each of our four models that fall outside ecologically realistic values. The proportion of predictions in the correct range increases with species richness for the constant connectance and power law models. Shaded area shows the 5%, 50%, and 95% quantiles of the distribution of S, demonstrating that many communities have potentially incorrect predictions under previous models.

that adding n species should increase the linkage density by approximately $p \times n$. The addition of 11 new species (p^{-1} according to Table 2) should increase the linkage density in the food web by roughly 1, meaning that each species in the original network would be expected to develop two additional interactions. Similarly, Equation 6 shows that when S is large, we should expect a connectance that is a constant. Thus, p has an interesting ecological interpretation: it represents the average connectance of networks large enough that the proportion $(S-1)/S^2$ is negligible.

Applications of the Flexible Links Model to Key Food Web Questions

Our model is generative, which is important and useful: we can use this model to correctly generate predictions that look like real data. This suggests that we can adapt the model, using either its parameters or predictions or both, to get a new perspective on many questions in network ecology. Here, we show four possible applications that we think are interesting, in that relying on our model eliminates the need to speculate on the structure of networks or to introduce new hypotheses to account for it.

Probability Distributions for L_D and Co

In a beta-binomial distribution, it is assumed that the probability of success p varies among groups of trials according to a Beta$(\mu\phi, (1-\mu)\phi)$ distribution. Since p has a beta distribution, the linear transformations described by Equations 6 and 7 also describe beta distributions that have been shifted and scaled according to the number of species S in a community. This shows that just as L must be within ecologically meaningful bounds, Co (Equation 6) and L_D (Equation 7) must be as well. The connectance of a food web is bounded by $(S-1)/S^2$ and 1, while the linkage density is bounded by $(S-1)/S$ and S.

We can convert the beta distribution for p into one for Co by replacing p with the transformation of Co as described above (Equation 6), and rescaling by the new range:

$$[\mathrm{Co}|S,\mu,\phi] = \frac{\left(\mathrm{Co}-\frac{S-1}{S^2}\right)^{\mu\phi-1}(1-\mathrm{Co})^{(1-\mu)\phi-1}}{\left(1-\frac{S-1}{S^2}\right)^{\phi-1}\times B(\mu\phi,\ (1-\mu)\phi)}. \quad \text{(Equation 8)}$$

Similarly, we can convert the distribution for p into one for L_D by replacing p with the transformation that gives L_D (Equation 7):

$$[L_D|S,\mu,\phi] = \frac{\left(L_D-\frac{S-1}{S}\right)^{\mu\phi-1}(1-L_D)^{(1-\mu)\phi-1}}{\left(S-\frac{S-1}{S}\right)^{\phi-1}\times B(\mu\phi,\ (1-\mu)\phi)}. \quad \text{(Equation 9)}$$

In Figure 3, we show that the connectance and linkage density obtained from the equations above fit the empirical data well.

An Analytic Alternative to Null-Model Testing

Ecologists are often faced with the issue of comparing several networks. A common question is whether a given network has an "unusual" number of links relative to some expectation. Traditionally these comparisons have been done by simulating a "null" distribution of random matrices.[24,25] This is intended to allow ecologists to compare food webs with a sort of standard, hopefully devoid of whatever biological process could alter the number of links. Importantly, this approach assumes that (1) connectance is a fixed property of the network, ignoring any stochasticity, and (2) the simulated network distribution is an accurate and unbiased description of the null distribution. Yet recent advances in the study of probabilistic ecological networks show that the existence of links, and connectance itself, is best thought of as a probabilistic quantity.[18] Given that connectance drives most of the measures of food web structure,[17] it is critical to have a reliable means of measuring differences from the expectation. We provide a way to assess whether the number of links in a network (and therefore its connectance) is surprising. We do so using mathematics rather than simulations.

Table 2. Parameter Estimates for all Models

Model	Parameter	Interpretation	Value	SD
bS (Cohen and Briand[11])	b	links per species	2.2	0.047
	κ	concentration of L around mean	1.4	0.12
bS^2 (Martinez[12])	b	proportion of links realized	0.12	0.0041
	κ	concentration of L around mean	4.0	0.37
bS^a (Brose et al.[13])	b	proportion of relationship	0.37	0.054
	a	scaling of relationship	1.7	0.043
	κ	concentration of L around mean	4.8	0.41
$(S^2-(S-1))p+S-1$	μ	average value of p	0.086	0.0037
	ϕ	concentration around value of μ	24.3	2.4

Mean and standard deviation (SD) are given for each parameter.

The shifted beta-binomial can be approximated by a normal distribution with mean $\bar{L}$ and variance σ_L^2:

$$L \sim \mathrm{Normal}\left(\bar{L}, \sigma_L^2\right)$$

$$\bar{L} = \left(S^2 - S + 1\right)\mu + S - 1$$

$$\sigma_L^2 = \left(S^2 - S + 1\right)\mu(1-\mu)\left(1 + \frac{S(S-1)}{\phi+1}\right). \quad \text{(Equation 10)}$$

This normal approximation is considered good whenever the skewness of the target distribution is modest. In food webs, this should be true whenever communities have more than about ten species (see Experimental Procedures). This result means that given a network with observed species richness S_{obs} and observed links L_{obs}, we can calculate its Z score, i.e., how many standard deviations an observation is from the population average, as

$$Z = \frac{L_{obs} - \bar{L}}{\sqrt{\sigma_L^2}}. \quad \text{(Equation 11)}$$

A network where $L = \bar{L}$ will have a Z score of 0, and any network with more (fewer) links will have a positive (negative) Z score. Following this method, we computed the empirical Z scores for the 255 food webs archived on mangal.io (Figure 4). We found that 18 webs (7.1%) had a total number of observed links unusually higher than what was expected under the flexible links model ($Z > 1.96$). These networks are interesting candidates for the study of mechanisms leading to high connectance.

Out of the 255 food webs, none was found to have an unusually low number of links ($Z < 1.96$). In fact, Z scores this low are not possible in this dataset: food webs having the minimum value of $S - 1$ links are still within two standard deviations of the mean for this sample. However, this sample contains the full diversity of food webs found in the mangal.io database. Hence, this does not mean that no food web will ever have a Z score lower than -1.96. If the flexible links model is fit to data from a specific system, food webs might have a surprisingly low number of links when compared with this population average. These networks would be interesting candidates for the study of mechanisms leading to low connectance or for the identification of undersampled webs. Ecologists can thus use our method to assess the deviation of their own food webs from their random expectations.

In Figure 5, we show that the predictions made by the normal approximation (Figure 5B) are similar to those made by the beta distribution parameterized with the MAP values of μ and ϕ (Figure 5A), although the former can undershoot the constraint on the minimum number of links. This undershooting, however, will not influence any actual Z scores, since no food webs have fewer than $S - 1$ links and therefore no Z scores so low can ever be observed.

We Should See Many Different Network-Area Relationships

Our results bear important consequences for the nascent field of studying network-area relationships.[26] As it has long been observed that not all species in a food web diffuse equally through space,[27] understanding how the shape of networks varies when the area increases is an important goal, and in fact underpins the development of a macroecological theory of food webs.[28] Using a power law as the acceptable relationship between species and area,[29,30] the core idea of studying network-area relationships is to predict network structure as a consequence of the effect of spatial scale on species richness.[26] Drawing on these results, we provide in Figure 6 a simple illustration of the fact that, due to the dispersal of values of L, the relationship between L/S and area can have a really wide confidence interval. While our posterior predictions generally match the empirical results on this topic,[31] they suggest that we will observe many relationships between network structure and space, and that picking out the signal of network-area relationships might be difficult.

As of now not many network-area relationships have been documented empirically; but after the arguments made by Galiana et al.[26] that tie the shape of these relationships to macroecological processes, we fully expect these relationships to be more frequently described moving forward. Our results suggest that our expectation of the amount of noise in these relationships should be realistic; while it is clear that these relationships exist, because of the scaling of dispersion in the number of links with the number of species, they will necessarily be noisy. Any described relationships will exist within extremely wide confidence intervals, and it might require a large quantity of empirical data to properly characterize them. As such, our model can help

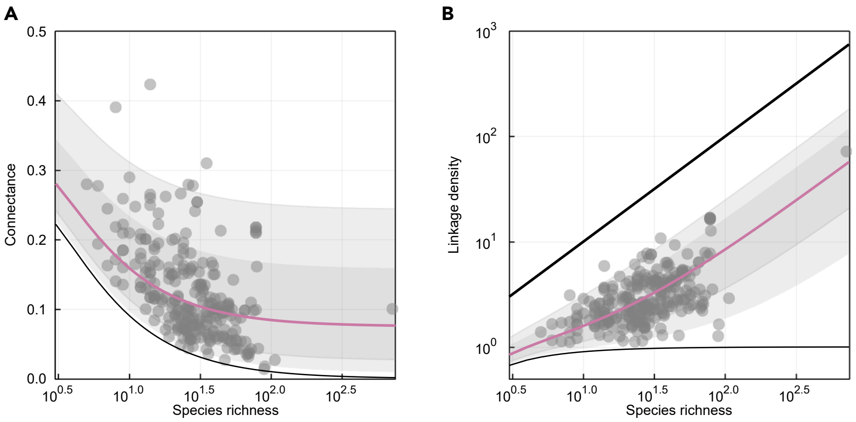

Figure 3. Connectance and Linkage Density Can Be Derived from a Model for Links

(A and B) (A) Connectance and (B) linkage density are plotted as a function of species richness, for the MAP estimates of the flexible links model. In each panel, the colored line represents the median predicted quantity and the gray areas cover the 78% and 97% percentile intervals. Empirical data from the mangal.io database are plotted in each panel (gray dots). In (A), the minimal $(S-1)/S^2$ connectance and in (B) the minimal $(S-1)/S$ and maximum S linkage density are plotted (black lines).

in assessing the difficulty of capturing some foundational relationships of food web structure.

Stability Imposes a Limit on Network Size

Can organisms really interact with an infinite number of partners? According to Equation 7, at large values of S, the linkage density scales according to $p\times S$ (which is supported by empirical data), and so species are expected to have on average $2\times p\times S$ interactions. A useful concept in evolutionary biology is the "Darwinian demon,"[32] i.e., an organism that would have infinite fitness in infinite environments. Our model seems to predict the emergence of what we call Eltonian demons, which can have an arbitrarily large number of interactions. Yet we know that constraints on handling time of prey, for example, impose hard limits on diet breadth.[33] This result suggests that there are other limitations to the size of food webs; indeed, the fact that L/S increases to worryingly large values only matters if ecological processes allow S to be large enough. It is known that food webs can reach as high as energy transfer allows[5] and as wide as competition allows.[34] Furthermore, in more species-rich communities there is a greater diversity of functional traits among the interacting organisms; this limits interactions, because traits determine suitable interaction partners.[35,36] In short, and as Figure 2 suggests, since food webs are likely to be constrained to remain within an acceptable richness, we have no reason to anticipate that $p\times S$ will keep growing infinitely.

Network structure may itself prevent S from becoming large. May[37] suggested that a network of richness S and connectance Co is stable as long as the criterion $\sigma\sqrt{S\times Co}<1$ is satisfied, with σ being the standard deviation of the strengths of interactions. Although this criterion is not necessarily stringent enough for the stability of food webs,[38,39] it still defines an approximate maximum value σ^* which is the value above which the system is expected to be unstable. This threshold is $\sigma^* = 1/\sqrt{L_D}$, where L_D is defined as in Equation 7. We illustrate this result in Figure 7, which reveals that σ^* falls toward 0 for larger species richness. The result in Figure 7 is in agreement with previous simulations,

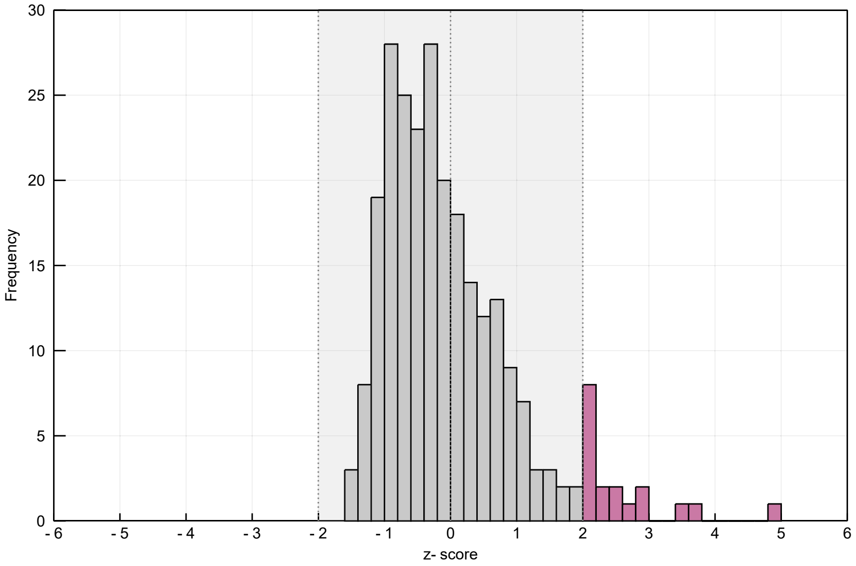

Figure 4. Empirical Distribution of Food Web *Z* scores

The *Z* scores of all food webs archived on mangal.io have been computed using Equation 11. Food webs with an absolute *Z* score above 1.96 are in pink. The shaded region comprises all food webs with an absolute *Z* score below 1.96 (i.e., 95% of predicted webs according to our model).

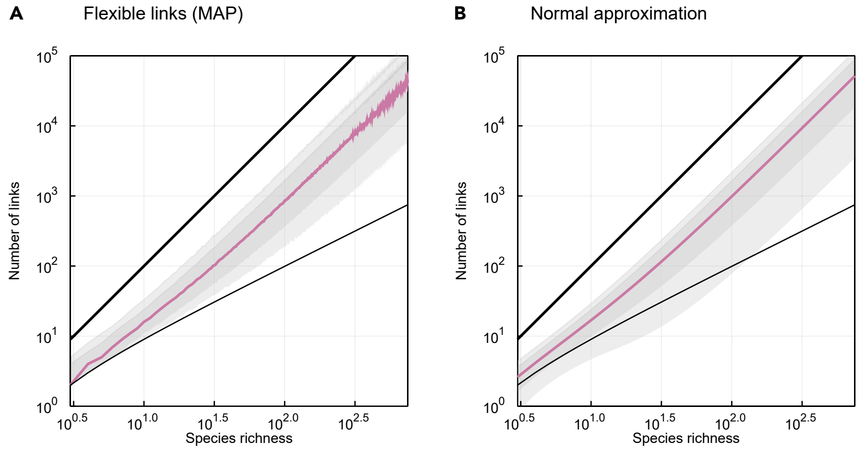

Figure 5. The Shifted Beta-Binomial Distribution Can Be Approximated by a Normal Distribution

The number of links is plotted as a function of species richness obtained from (A) the MAP estimates of the flexible links model and (B) its normal approximation. In each panel, the colored line represents the median predicted link number and the gray areas cover the 78% and 97% percentile intervals. The minimal $S-1$ and maximal S^2 numbers of links are plotted in each panel (thinner and bolder black lines, respectively).

placing the threshold for stability at about 1,200 species in food webs. These results show how ecological limitations, for example on connectance and the resulting stability of the system, can limit the size of food webs.[38,40] In Figure 7B, we show that networks of increasing richness (thicker lines, varying on a log scale from 10^1 to 10^3) have a lower probability of being stable, based on the proportion of stable networks in our posterior samples.

Conclusions

Here we derived Equation 4, a model for the prediction of the number of links in ecological networks using a beta-binomial distribution for L, and show how it outperforms previous and more commonly used models describing this relationship. More importantly, we showed that our model has parameters with a clear ecological interpretation (specifically, the value of p in Equation 4 is the expected value of the connectance when S is large), and makes predictions that remain within biological boundaries. There are a variety of "structural" models for food webs, such as the niche model,[41] the cascade model,[42] the diet breadth model (DBM)[35] and allometric DBM,[19] the minimum potential niche model,[43] and the nested hierarchy model,[44] to name a few. All of these models make predictions of food web structure: based on some parameters (usually S and L, and sometimes vectors of species-level parameters) they output an adjacency matrix $A_{S\times S}$, which contains either the presence or strength of trophic interactions. Therefore, these models require estimated values of L for a particular value of S, with the additional result that $\sum A = L$. Our approach can serve to improve the realism of these models by imposing that the values of L they use are within realistic boundaries. For example, a common use of structural models is to generate a set of "null" predictions: possible values of A and L in the absence of the mechanism of interest. Empirical networks are then compared with this set of predictions and are said to be significant if they are more extreme than 95% of the observations.[3] A challenge in this approach is that structural models may generate a wide range of predictions, including ecologically impossible values, leading a high false-negative rate. This could be remedied by filtering this set of predictions according to our flexible links model, resulting in a narrower set of null predictions and a lower false-negative rate. In general, our approach is complementary to other attempts to create ecologically realistic food web models—for example, probabilistic models of the number of links per species which stay within ecological values.[45]

This model also casts new light on previous results on the structure of food webs: small and large food webs behave differently.[15] Specifically, ecological networks most strongly deviate from scale-free expectations when connectance is high.[46] In our model, this behavior emerges naturally: connectance increases sharply as species richness decreases (Figure 3)—that is, where the additive term $(S-1)/S^2$ in Equation 6 becomes progressively larger. In a sense, small ecological networks are different only due to the low values of S. Small networks have only a very limited number of flexible links, and this drives connectance to be greater. Connectance in turn has implications for many ecological properties. Connectance is more than the proportion of realized interactions. It has been associated with some of the most commonly used network measures[17] and contains meaningful information on the stability[46,47] and dynamics[48] of ecological communities. A probability distribution for connectance not only accounts for the variability between networks but can be used to describe fundamental properties of food webs and to identify ecological and evolutionary mechanisms that shape communities. A recent research direction has been to reveal its impact on resistance to invasion: denser networks with a higher connectance are comparatively more difficult to invade;[49] different levels of connectance are also associated with different combinations of primary producers, consumers, and apex predators,[41] which in turns determines which kind of species will have more success invading the network.[50] Because we can infer connectance from the richness of a community, our model also ties the invasion resistance of a network to its species richness.

The relationship between L and S has underpinned most of the literature on food web structure since the 1980s. Additional generations of data have allowed us to progress from the link-species scaling law, to constant connectance, to more general formulations based on a power law. Our model breaks with this tradition of iterating over the same family of relationships, and instead draws from our knowledge of ecological processes and from novel tools in probabilistic programming. As a result, we provide predictions of the number of links that are closer to empirical data, stimulate new ecological insights, and can be safely assumed to always fall within realistic values. The results

CellPress
OPEN ACCESS

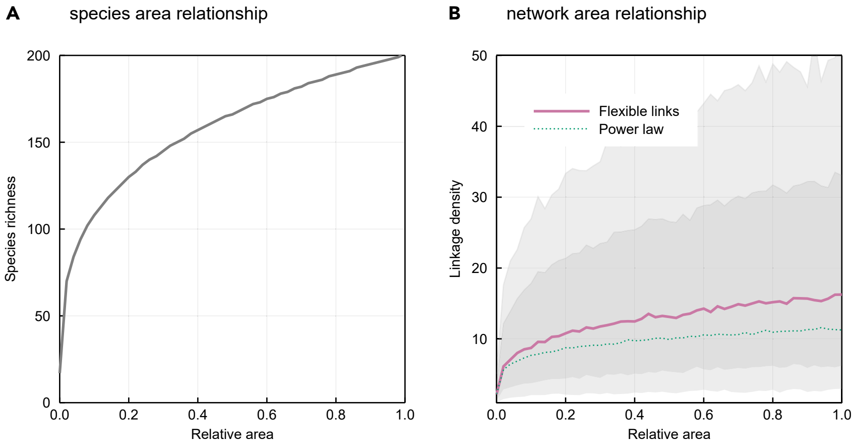

Figure 6. Many Different Network-Area Relationships Are Supported by the Data

Representing the species richness as $S = kA^z$ (A), with A being the relative area size, $k = 200$ being the maximal species richness, and $z = 0.27$, a scaling exponent.[26] We then use the posterior distribution of L to predict how L_D should scale with A. We compare the predictions of our model with that of the generally accepted power law (Equation 3). While our model predicts a larger linkage density in larger areas (B), the confidence intervals around this prediction (gray areas covering the 78% and 97% percentile intervals) are extremely large. In particular, our model scales faster than the power law, but the confidence interval is high (due to the scaling of variance with S, Equation 10). This suggests that we may observe either very weak or very strong effects of area on networks.

presented in Figure 6 (which reproduces results from Galiana et al.[26]) and Figure 7 (which reproduces results from Allesina and Tang[38]) may seem largely confirmatory; in fact, the ability of our model to reach the conclusions of previous milestone studies in food web ecology is a strong confirmation of its validity. We would like to point out that these approaches would usually require ecologists to make inferences not only on the parameters of interests but also on the properties of a network for a given species richness. In contrast, our model allows a real economy of parameters and offers ecologists the ability to obtain several key elements of network structure for free if only the species richness is known.

EXPERIMENTAL PROCEDURES

Resource Availability

Lead Contact

Further information and requests for resources and code should be directed to and will be fulfilled by the Lead Contact, Arthur Andrew Meahan MacDonald (a.a.m.macdonald@gmail.com).

Materials Availability

This study did not generate new unique reagents.

Data and Code Availability

All code and data to reproduce this article are available at the Open Science Framework (https://doi.org/10.17605/OSF.IO/YGPZ2).

Bayesian Model Definitions

Generative models are flexible and powerful tools for understanding and predicting natural phenomena. These models aim to create simulated data with the same properties as observations. Creating such a model involves two key components: a mathematical expression, which represents the ecological process being studied, and a distribution, which represents our observations of this process. Both of these components can capture our ecological understanding of a system, including any constraints on the quantities studied.

Bayesian models are a common set of generative models, frequently used to study ecological systems. Here, we define Bayesian models for all four of the models described in Equations 1, 2, 3, and 4. We use notation from Hobbs and Hooten,[51] writing out both the likelihood and the prior as a product over all 255 food webs in the mangal.io database.

Link-Species Scaling Model

$$[b, \kappa | \mathbf{L}, \mathbf{S}] \propto \prod_{i=1}^{255} \text{negative binomial}(L_i | b \times S_i, e^{\kappa}) \times \text{normal}(b | 0.7, 0.02) \times \text{normal}(\kappa | 2, 1)$$

Constant Connectance Model

$$[b, \kappa | \mathbf{L}, \mathbf{S}] \propto \prod_{i=1}^{255} \text{negative binomial}\left(L_i | b \times S_i^2, e^{\kappa}\right) \times \text{beta}(b | 3, 7) \times \text{normal}(\kappa | 2, 1)$$

Power Law Model

$$[b, a, \kappa | \mathbf{L}, \mathbf{S}] \propto \prod_{i=1}^{255} \text{negative binomial}\left(L_i | \exp(b) \times S_i^a, e^{\kappa}\right) \times \text{normal}(b | -3, 1) \times \text{normal}(a | 2, 0.6) \times \text{normal}(\kappa | 2, 1)$$

Flexible Links Model

$$[\mu, \phi | \mathbf{L}, \mathbf{S}] \propto \prod_{i=1}^{255} \text{beta binomial}\left(L_i - S_i + 1 | S_i^2 - S_i + 1, \mu \times e^{\phi}, (1 - \mu) \times e^{\phi}\right) \times \text{beta}(\mu | 3, 7) \times \text{normal}(\phi | 3, 0.5)$$

Note that while e^{ϕ} is shown in these equations for clarity, in the text we use ϕ to refer to the parameter after exponentiation. In the above equations, bold type indicates a vector of values; we use capital letters for L and S for consistency with the main text.

Because we want to compare all our models using information criteria, we were required to use a discrete likelihood to fit all models. Our model uses a discrete likelihood by default, but the previous three models (LSSL, constant connectance, and the power law) normally do not. Instead, these models have typically been fit with Gaussian likelihoods, sometimes after log-transforming L and S. For example, Equation 3 becomes a linear relationship between $\log(L)$ and $\log(S)$. This ensures that predictions of L are always positive, but allows otherwise unconstrained variation on both sides of the mean. To keep this same spirit, we chose the negative binomial distribution for observations. This distribution is limited to positive integers and can vary on both sides of the mean relationship.

We selected priors for our Bayesian models using a combination of literature and domain expertise. For example, we chose our prior distribution for p based on Martinez,[12] who gave a value of constant connectance equal to 0.14. While the prior we use is "informative," it is weakly so; as Martinez[12] did not provide an estimate of the variance for his value, we chose a relatively large variation around that mean. However, no information is available in the literature to inform a choice of prior for concentration parameters κ and ϕ. For these values, we followed the advice of Gabry et al.[52] and performed prior predictive checks. Specifically, we chose priors that generated a wide range of values for L_i but which did not frequently predict webs of either maximum or minimum connectance, neither of which are observed in nature.

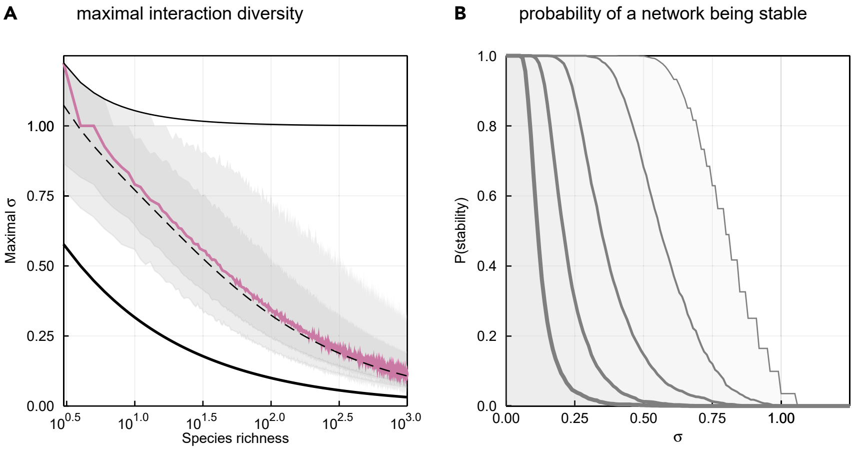

Figure 7. Stability Imposes a Limit on Network Size

Using Equation 7, we can calculate the maximum standard deviation in the strength of interactions that should ensure food web stability, $\sigma^* = 1/\sqrt{L_D}$ (A). The colored line represents the median value of maximum standard deviation, based on the posterior distribution of the flexible links model, and the gray areas cover the 78% and 97% percentile intervals. The fine and dark lines indicate the maximum and minimum values of maximum standard deviation, respectively. The dotted line shows the maximum for the average L_D, as given by Equation 7. The maximum standard deviation falls sharply when the number of species increases, which will limit the stability of large food webs, and therefore explain why Eltonian demons should not emerge. In (B), we show the probability of a network with S species being stable, based on draws from the posterior distribution, for $10 \leq S \leq 1{,}000$: larger networks (thicker lines) are increasingly unlikely to be stable.

Explanation of Shifted Beta-Binomial Distribution

Equation 4 implies that L_{FL} has a binomial distribution, with $S^2 - S + 1$ trials and a probability p of any flexible link being realized:

$$[L|S,p] = \binom{S^2-(S-1)}{L-(S-1)} p^{L-(S-1)}(1-p)^{S^2-L}.$$

This is often termed a shifted binomial distribution.

We also note that ecological communities are different in many ways besides their number of species (S). Although we assume p to be fixed within one community, the precise value of p will change from one community to another. With this assumption, our likelihood becomes a shifted beta-binomial distribution:

$$[L|S,\mu,\phi] = \binom{S^2-(S-1)}{L-(S-1)} \frac{B\left(L-(S-1)+\mu\phi, S^2-L+(1-\mu)\phi\right)}{B(\mu\phi,(1-\mu)\phi)},$$

(Equation 12)

where B is the beta function. Thus, the problem of fitting this model becomes one of estimating the parameters of this univariate probability distribution.

Model Fitting: Data and Software

We evaluated our model against 255 empirical food webs, available in the online database mangal.io.[21] We queried metadata (number of nodes and number of links) for all networks, and considered as food webs all networks having interactions of predation and herbivory. We use Stan,[53] which implements Bayesian inference using Hamiltonian Monte Carlo. We ran all models using four chains and 2,000 iterations per chain. In our figures we use the posterior predictive distribution, which is a distribution described by the model after conditioning on the data. There are numerous ways to display a probability distribution; here we have chosen to do so using the expectation (mean) and two arbitrary percentile intervals, 78% and 97%. These intervals were chosen based on the recommendations of McElreath,[54] and allowed us to capture most of the probability density in the tails of the posterior distributions.

Stan provides a number of diagnostics for samples from the posterior distribution, including $\widehat{R}$, effective sample size, and measures of effective tree depth and divergent iterations. None of these indicated problems with the posterior sampling. All models converged with no warnings; this indicates that is it safe to make inferences about the parameter estimates and to compare the models. However, the calculation of PSIS-LOO for the LSSL model warned of problematic values of the Pareto-k diagnostic statistic. This indicates that the model is heavily influenced by large values. Additionally, we had to drop the largest observation (>50,000 links) from all datasets in order to calculate PSIS-LOO for the LSSL model. Taken together, this suggests that the LSSL model is insufficiently flexible to accurately reproduce the data.

Normal Approximation and Analytic *Z* Scores

We propose using a normal approximation to the beta-binomial distribution to calculate analytic Z scores. This is based on a well-known similarity between the shape of a normal distribution and a binomial distribution. This approximation is considered good whenever the absolute skewness is less than 0.3,[55] that is, whenever

$$\frac{1}{\sqrt{S^2-S+1}}\left(\sqrt{\frac{1-\mu}{\mu}}-\sqrt{\frac{\mu}{1-\mu}}\right)<0.3.$$

The beta-binomial distribution is close to the binomial distribution. The error in approximating the former with the latter is on the order of the inverse square of the parameter ϕ,[56] which for our model is less than 0.0017.

SUPPLEMENTAL INFORMATION

Supplemental Information can be found online at https://doi.org/10.1016/j.patter.2020.100079.

ACKNOWLEDGMENTS

We thank three anonymous reviewers, as well as Matthew Barbour, Owen Petchey, and Andrew Beckerman, for their helpful comments and suggestions. Funding was provided by the Institute for Data Valorization (IVADO) and the NSERC CREATE Computational Biodiversity Science and Services ($BIOS^2$) training program. T.P. acknowledges funding from the John Evans Leaders' funds from the Canada Foundation for Innovation. This work was made possible thanks to the effort of volunteers developing high-quality free and open-source software for research.

AUTHOR CONTRIBUTIONS

Conceptualization, A.A.M.M., F.B., and T.P.; Methodology, A.A.M.M., F.B., and T.P.; Software, A.A.M.M., F.B., and T.P.; Validation, A.A.M.M., F.B., and T.P.; Formal analysis, A.A.M.M., F.B., and T.P.; Investigation, A.A.M.M., F.B., and T.P.; Data Curation, T.P.; Writing – Original Draft, A.A.M.M., F.B., and T.P.; Writing – Review & Editing, A.A.M.M., F.B., and T.P.; Visualization, A.A.M.M., F.B., and T.P.; Supervision, A.A.M.M. and T.P.

DECLARATION OF INTERESTS

The authors declare no competing interests.

Received: February 18, 2020
Revised: April 7, 2020
Accepted: July 3, 2020
Published: July 29, 2020

REFERENCES

1. Borrett, S.R., Moody, J., and Edelmann, A. (2014). The rise of Network Ecology: maps of the topic diversity and scientific collaboration. Ecol. Model. *293*, 111–127.
2. Canard, E., Mouquet, N., Marescot, L., Gaston, K.J., Gravel, D., and Mouillot, D. (2012). Emergence of structural patterns in neutral trophic networks. PLOS ONE *7*, e38295.
3. Delmas, E., Besson, M., Brice, M.-H., Burkle, L.A., Dalla Riva, G.V., Fortin, M.-J., Gravel, D., Guimarães, P.R., Hembry, D.H., Newman, E.A., et al. (2018). Analysing ecological networks of species interactions. Biol. Rev. *94*, 16–36.
4. McCann, K.S. (2012). Food Webs (Princeton University Press).
5. Thompson, R.M., Brose, U., Dunne, J.A., Hall, R.O., Hladyz, S., Kitching, R.L., Martinez, N.D., Rantala, H., Romanuk, T.N., Stouffer, D.B., et al. (2012). Food webs: reconciling the structure and function of biodiversity. Trends Ecol. Evol. *27*, 689–697.
6. McCann, K. (2007). Protecting biostructure. Nature *446*, 29.
7. Seibold, S., Cadotte, M.W., MacIvor, J.S., Thorn, S., and Müller, J. (2018). The necessity of multitrophic approaches in community ecology. Trends Ecol. Evol. *33*, 754–764.
8. Jordano, P. (2016). Sampling networks of ecological interactions. Funct. Ecol. *30*, 1883–1893.
9. Jordano, P. (2016). Chasing ecological interactions. PLOS Biol. *14*, e1002559.
10. Pascual, M., and Dunne, J.A. (2006). Ecological Networks: Linking Structure to Dynamics in Food Webs (Oxford University Press).
11. Cohen, J.E., and Briand, F. (1984). Trophic links of community food webs. Proc. Natl. Acad. Sci. U S A *81*, 4105–4109.
12. Martinez, N.D. (1992). Constant connectance in community food webs. Am. Nat. *139*, 1208–1218.
13. Brose, U., Ostling, A., Harrison, K., and Martinez, N.D. (2004). Unified spatial scaling of species and their trophic interactions. Nature *428*, 167–171.
14. Winemiller, K., Pianka, E., Vitt, L., and Joern, A. (2001). Food web laws or niche theory? six independent empirical tests. Am. Nat. *158*, 193–199.
15. Garlaschelli, D., Caldarelli, G., and Pietronero, L. (2003). Universal scaling relations in food webs. Nature *423*, 165–168.
16. Riede, J.O., Rall, B.C., Banasek-Richter, C., Navarrete, S.A., Wieters, E.A., Emmerson, M.C., Jacob, U., and Brose, U. (2010). Chapter 3: scaling of food-web properties with diversity and complexity across ecosystems. In Advances in Ecological Research Ecological Networks, G. Woodward, ed. (Academic Press), pp. 139–170.
17. Poisot, T., and Gravel, D. (2014). When is an ecological network complex? Connectance drives degree distribution and emerging network properties. PeerJ *2*, e251.
18. Poisot, T., Cirtwill, A.R., Cazelles, K., Gravel, D., Fortin, M.-J., and Stouffer, D.B. (2016). The structure of probabilistic networks. Methods Ecol. Evol. *7*, 303–312.
19. Petchey, O.L., Beckerman, A.P., Riede, J.O., and Warren, P.H. (2008). Size, foraging, and food web structure. Proc. Natl. Acad. Sci. U S A *105*, 4191–4196.
20. Poisot, T., Stouffer, D.B., and Gravel, D. (2015). Beyond species: why ecological interaction networks vary through space and time. Oikos *124*, 243–251.
21. Poisot, T., Baiser, B., Dunne, J.A., Kéfi, S., Massol, F., Mouquet, N., Romanuk, T.N., Stouffer, D.B., Wood, S.A., and Gravel, D. (2016). Mangal—making ecological network analysis simple. Ecography *39*, 384–390.
22. Poisot, T., Bergeron, G., Cazelles, K., Dallas, T., Gravel, D., Macdonald, A., Mercier, B., Violet, C., and Vissault, S. (2020). Environmental biases in the study of ecological networks at the planetary scale. bioRxiv. https://doi.org/10.1101/2020.01.27.921429.
23. Vehtari, A., Gelman, A., and Gabry, J. (2017). Practical Bayesian model evaluation using leave-one-out cross-validation and WAIC. Stat. Comput. *27*, 1413–1432.
24. Fortuna, M.A., and Bascompte, J. (2006). Habitat loss and the structure of plant-animal mutualistic networks: mutualistic networks and habitat loss. Ecol. Lett. *9*, 281–286.
25. Bascompte, J., Jordano, P., Melian, C.J., and Olesen, J.M. (2003). The nested assembly of plant-animal mutualistic networks. Proc. Natl. Acad. Sci. U S A *100*, 9383–9387.
26. Galiana, N., Lurgi, M., Claramunt-López, B., Fortin, M.-J., Leroux, S., Cazelles, K., Gravel, D., and Montoya, J.M. (2018). The spatial scaling of species interaction networks. Nat. Ecol. Evol. *2*, 782–790.
27. Holt, R.D., Lawton, J.H., Polis, G.A., and Martinez, N.D. (1999). Trophic rank and the species-area relationship. Ecology *80*, 1495–1504.
28. Baiser, B., Gravel, D., Cirtwill, A.R., Dunne, J.A., Fahimipour, A.K., Gilarranz, L.J., Grochow, J.A., Li, D., Martinez, N.D., McGrew, A., et al. (2019). Ecogeographical rules and the macroecology of food webs. Glob. Ecol. Biogeogr. *28*, 1204–1218.
29. Dengler, J. (2009). Which function describes the species-area relationship best? A review and empirical evaluation. J. Biogeogr. *36*, 728–744.
30. Williamson, M., Gaston, K.J., and Lonsdale, W.M. (2001). The species-area relationship does not have an asymptote! J. Biogeogr. *28*, 827–830.
31. Wood, S.A., Russell, R., Hanson, D., Williams, R.J., and Dunne, J.A. (2015). Effects of spatial scale of sampling on food web structure. Ecol. Evol. *5*, 3769–3782.
32. Law, R. (1979). Optimal life histories under age-specific predation. Am. Nat. *114*, 399–417.
33. Forister, M.L., and Jenkins, S.H. (2017). A neutral model for the evolution of diet breadth. Am. Nat. *190*, E40–E54.
34. Kéfi, S., Berlow, E.L., Wieters, E.A., Navarrete, S.A., Petchey, O.L., Wood, S.A., Boit, A., Joppa, L.N., Lafferty, K.D., Williams, R.J., et al. (2012). More than a meal… integrating non-feeding interactions into food webs. Ecol. Lett. *15*, 291–300.
35. Beckerman, A.P., Petchey, O.L., and Warren, P.H. (2006). Foraging biology predicts food web complexity. Proc. Natl. Acad. Sci. U S A *103*, 13745–13749.
36. Bartomeus, I., Gravel, D., Tylianakis, J.M., Aizen, M.A., Dickie, I.A., and Bernard-Verdier, M. (2016). A common framework for identifying linkage rules across different types of interactions. Funct. Ecol. *30*, 1894–1903.
37. May, R.M. (1972). Will a large complex system be stable? Nature *238*, 413–414.
38. Allesina, S., and Tang, S. (2012). Stability criteria for complex ecosystems. Nature *483*, 205–208.
39. Allesina, S., and Tang, S. (2015). The stability-complexity relationship at age 40: a random matrix perspective. Popul. Ecol. *57*, 63–75.
40. Borrelli, J.J., Allesina, S., Amarasekare, P., Arditi, R., Chase, I., Damuth, J., Holt, R.D., Logofet, D.O., Novak, M., Rohr, R.P., et al. (2015). Selection on stability across ecological scales. Trends Ecol. Evol. *30*, 417–425.
41. Williams, R.J., and Martinez, N.D. (2000). Simple rules yield complex food webs. Nature *404*, 180–183.
42. Cohen, J.E., and Newman, C.M. (1985). A stochastic theory of community food webs I. Models and aggregated data. Proc. R. Soc. Lond. B *224*, 421–448.
43. Allesina, S., Alonso, D., and Pascual, M. (2008). A general model for food web structure. Science *320*, 658–661.
44. Cattin, M.-F., Bersier, L.-F., Banašek-Richter, C., Baltensperger, R., and Gabriel, J.-P. (2004). Phylogenetic constraints and adaptation explain food-web structure. Nature *427*, 835–839.
45. Kozubowski, T.J., Panorska, A.K., and Forister, M.L. (2015). A discrete truncated Pareto distribution. Stat. Methodol. *26*, 135–150.
46. Dunne, J.A., Williams, R.J., and Martinez, N.D. (2002). Food-web structure and network theory: the role of connectance and size. Proc. Natl. Acad. Sci. U S A *99*, 12917–12922.

47. Montoya, J.M., Pimm, S.L., and Solé, R.V. (2006). Ecological networks and their fragility. Nature *442*, 259–264.

48. Vieira, M.C., and Almeida-Neto, M. (2015). A simple stochastic model for complex coextinctions in mutualistic networks: robustness decreases with connectance. Ecol. Lett. *18*, 144–152.

49. Smith-Ramesh, L.M., Moore, A.C., and Schmitz, O.J. (2016). Global synthesis suggests that food web connectance correlates to invasion resistance. Glob. Change Biol. *23*, 465–473.

50. Baiser, B., Russell, G.J., and Lockwood, J.L. (2010). Connectance determines invasion success via trophic interactions in model food webs. Oikos *119*, 1970–1976.

51. Hobbs, N.T., and Hooten, M.B. (2015). Bayesian Models: A Statistical Primer for Ecologists (Princeton University Press).

52. Gabry, J., Simpson, D., Vehtari, A., Betancourt, M., and Gelman, A. (2019). Visualization in Bayesian workflow. J. R. Stat. Soc. A. *182*, 389–402.

53. Carpenter, B., Gelman, A., Hoffman, M.D., Lee, D., Goodrich, B., Betancourt, M., Brubaker, M., Guo, J., Li, P., and Riddell, A. (2017). Stan: a probabilistic programming language. J. Stat. Softw. *76*, 1–32.

54. McElreath, R. (2020). Statistical Rethinking: A Bayesian Course with Examples in R and STAN (CRC Press).

55. Box, G.E.P., Hunter, J.S., and Hunter, W.G. (2005). Statistics for Experimenters: Design, Innovation, and Discovery (Wiley-Interscience).

56. Teerapabolarn, K. (2014). An improved binomial approximation for the beta binomial distribution. Int. J. Pure Appl. Math. *97*, https://doi.org/10.12732/ijpam.v97i4.10.

PATTER, Volume *1*

Supplemental Information

Revisiting the Links-Species Scaling Relationship in Food Webs

Arthur Andrew Meahan MacDonald, Francis Banville, and Timothée Poisot

Supplemental Experimental Procedures

Parameter estimation by Maximum Likelihood

While the full posterior distribution can be sampled using various bayesian machinery, this is not necessary for obtaining point estimates of μ and ϕ. A maximum likelihood estimate of each can be calculated by rearranging eq. 4 and fitting a Beta distribution to the result.

We include this result because ecologists may wish to apply our methods for estimating L, Co or L/S without fitting a Bayesian posterior of their own. This approach loses information about the sample size of webs, but nevertheless provides a close match to both the empirical data and the bayesian posterior.

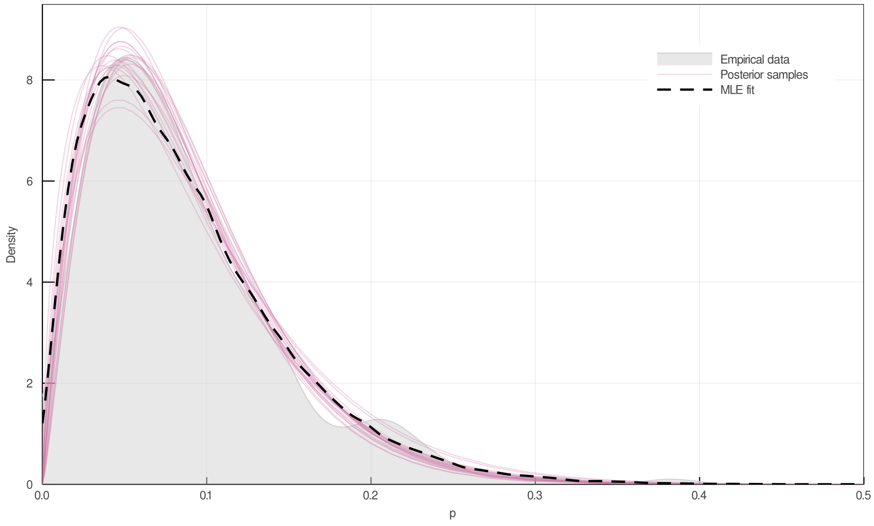

Figure 1: **Figure S1. Parameters can be estimated by Maximum Likelihood.** The maximum likelihood estimate of p is compared to 20 samples from the posterior distribution of the flexible links model. The empirical distribution of p, obtained from all food webs archived on the `mangal.io` database, is also included.

Table S1: Comparison between parameter estimates using maximum likelihood as described above (MLE estimate) and the maximum *a posteriori* values (MAP estimate) as reported in the main text.

parameter	MLE estimate	MAP estimate
μ	0.087	0.086 ± 0.0037

parameter	MLE estimate	MAP estimate
ϕ	21.0	24.3 $\pm$ 2.4
